# Inferring the Temporal Order of Cancer Gene Mutations in Individual Tumor Samples

**DOI:** 10.1371/journal.pone.0089244

**Published:** 2014-02-27

**Authors:** Jun Guo, Hanliang Guo, Zhanyi Wang

**Affiliations:** 1 School of Information and Communication Engineering, Beijing University of Posts and Telecommunications, Beijing, China; 2 Department of Aerospace and Mechanical Engineering, Viterbi School of Engineering, University of Southern California, Los Angeles, California, United States of America; 3 Department of Industry and Market Research China Mobile Research Institute, Beijing, China; Memorial Sloan Kettering Cancer Center, United States of America

## Abstract

The temporal order of cancer gene mutations in tumors is essential for understanding and treating the disease. Existing methods are unable to infer the order of mutations that are identified at the same time in individual tumor samples, leaving the heterogeneity of the order unknown. Here, we show that through a complex network-based approach, which is based on the newly defined statistic –*carcinogenesis information conductivity* (CIC), the temporal order in individual samples can be effectively inferred. The results suggest that tumor-suppressor genes might more frequently initiate the order of mutations than oncogenes, and every type of cancer might have its own unique order of mutations. The initial mutations appear to be dedicated to acquiring the function of evading apoptosis, and some order constraints might reflect potential regularities. Our approach is completely data-driven without any parameter settings and can be expected to become more effective as more data will become available.

## Introduction

Cancer is a genetic disease caused by the mutation of cancer genes consisting of oncogenes and tumor-suppressor genes. In most cancer cases, multiple mutations occur in a procedure known as tumor progression [1], [2]. To understand tumor progression, studies have been performed to model general regularities on the temporal order of mutations for a given type of cancer using both experimental and computational approaches [3]–[7]. As a canonical model, the order of mutations for colorectal cancer was reconstructed through tumor size and grade [8]. The latest computational models infer the typical temporal order constraints for certain type of cancers by simulating tumor progression as a stochastic process [9]–[11]. Despite this progress, there is still no well-defined method to infer the order of mutations identified at the same time in individual samples, although this inference is necessary to reveal the heterogeneity of the order of mutations in a cancer. Recently, as new generation sequencing becomes widely applied, the mutation landscapes in various cancers are being revealed one by one. The results have shown that the mutations in a cancer frequently demonstrate statistical correlations with each other or even cause-and-effect linkages of induction between the former and the latter [12]–[18]. However, these correlations/linkages have not been fully exploited in inferring the temporal order of mutations.

From an informatic perspective, this study defines a statistical measurement to assign value to the correlations or linkages mentioned above and model the mutations within a complex network, through which the temporal order of the mutations in individual samples can be inferred. We call the measurement the *carcinogenesis information conductivity* (CIC), which measures the reachability of transferring the information of a cancer gene having mutated to the transcription process of a given un-mutated cancer gene to induce its mutation. Statistically, the reachability can be estimated by the individual occurrence frequencies and the sequential co-occurrence frequency of the two genes' mutations in cancer samples. Additionally, competition among the information sent from multiple mutated genes to the given un-mutated gene should also be considered as any successful sending will cause the target gene to mutate, thus ending the mutation process. In this study, we call any two mutations found out in the same cancer sample co-occurrent mutations. While most genomic studies provide this quantity in an indirect way, here we aim at disentangling the sequence of occurrence of two mutational events from the simple co-occurrence. From these sequences of mutation occurrence, the sequential co-occurrence frequency can be calculated (Materials and Methods). Based on this idea, we have defined the CIC from cancer gene *i* to cancer gene *j* as: 
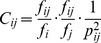
(1)where 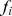 (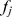) is the occurrence frequency of the mutation of gene *i* (*j*) in cancers, 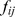 is the sequential co-occurrence frequency of the mutation of gene *i* followed by the mutation of gene *j*, and 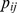 is the priority of gene *i* compared to other mutant genes to send the information to gene *j*. We have determined that 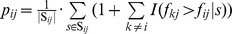. In this equation 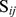 is the set of cancer samples with mutant genes *i* and *j*, 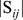 is the number of samples in the set, and 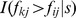 is an indicator function that equals 1 if 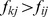 for the mutant genes 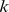, *j* and *i* in sample 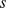. Otherwise it equals 0. Accordingly, the highest priority of one will be assigned if 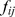 is larger than 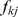 in every sample of the set, and the more times that 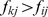, the larger value the 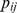. We regard formula (1) as a measurement of carcinogenesis information conductivity because the ratio 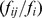 is an estimate of the maximum chance that gene *i* sends carcinogenesis information to gene *j* and causes its mutation, the ratio 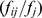 is an estimate of the maximum chance that the mutation of gene *j* is caused by carcinogenesis information received from gene *i*, and 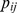 is the priority of the communication link compared with other links to gene *j*. The value of 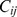 ranges from 0 to 1. Like the definition of *activation force*, a measurement we previously proposed for weighting the links of complex networks [19], the definition of CIC follows the formula of gravity if we imagine the ratios 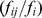 and 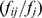 as masses and the priority 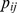 as distance. Statistics defined in this manner are likely to distribute their values in a power law, which is convenient for analyzing complex networks of intricate relationships including those in biology [20]–[24].

One challenge in computing the CICs is the lack of cancer samples that can be used as the source of the sequential co-occurrence frequencies of the cancer gene mutations because the mutations of different genes in a cancer sample are usually identified at the same time by sequencing. To tackle this challenge, we present an iterative procedure that couples CIC computation and the inference of the probability of every potential order of cancer gene mutation. The application of this procedure to the Catalogue of Somatic Mutations in Cancer (COSMIC) database [25], [26] revealed that the iteration reached convergence within fewer than 10 loops, and the convergent results suggest significant conclusions.

## Materials and Methods

### Iterative inference scheme

**Figure 1:**
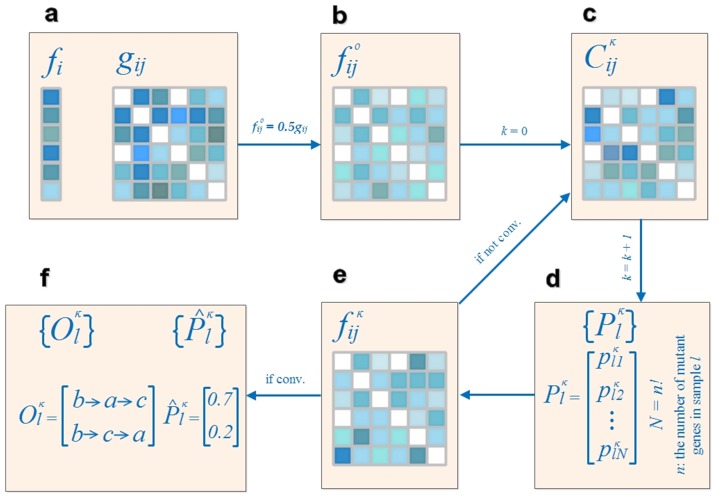
Overview of the inference methodology. (**a**) The occurrence and co-occurrence frequencies of the cancer gene mutations 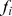 and 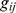 are determined from available samples, where 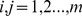, and 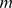 is the number of the cancer genes targeted in the study. An occurrence of a gene will be counted if it is mutated in one of the samples, and a co-occurrence of a pair of genes will be counted if both are mutated in one of the samples; therefore, 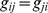 and 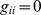. (**b**) Based on the principle of maximum entropy, the initial values of the sequential co-occurrence frequencies are set as 
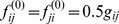
. (**c**) The carcinogenesis information conductivities, 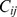, are calculated from the vector of 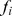 and the matrix of 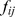. It should be noted that 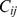 might not be equal to 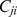, implying that the matrix of 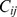 represents a directed network. (**d**) For each of the 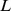 samples in question, the probabilities of every potential order of the mutant genes in sample 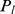 are computed according to the CICs of each order (Methods). (**e**) The matrix of 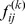 is redetermined by the matrix of 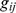 and the ratio of the probability-weighted number of the orders indicated that *i* occurs before *j* to the number of co-occurrence frequency, it is important to note that 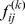 is not equal to 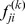 in general. If the matrix of 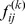 has not reached the criterion of convergence, the inferred orders will not be regarded as stable and a new loop of the calculation of 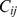 and 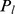 will be performed. Otherwise (**f**), the orders with a probability higher than random chance and the corresponding probabilities 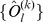 and 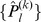 are regarded as the referred results. For example, of all 6 potential orders for a sample with three mutant cancer genes *a*, *b* and *c*, orders 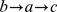 and 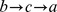 are identified as the probable ones due to probabilities of 0.7 and 0.2 (higher than a random chance of 1/6).

To perform the iterative inference procedure, a large set of cancer samples with cancer gene mutations identified by genome-wide sequencing is necessary. With the dataset, we determine the basic statistics of occurrence and non-sequential co-occurrence frequencies of cancer gene mutations. From these basic statistics, the iterative inference for the number of samples in question begins and the CIC results and probable orders of cancer gene mutation for each sample in question are determined when the iteration reaches convergence. Fig. 1 illustrates an overview of the procedure.

### Iterative procedure of CIC computation and inference of mutation order

By definition, sequential co-occurrence frequencies are necessary to estimate the CIC value. However, this requirement cannot be satisfied by the current databases, including COSMIC. To overcome this difficulty, we adopt an iterative procedure to couple the inference of the occurring mutation orders and the computation of the CICs. First, we evenly divide a non-sequential co-occurrence frequency into the two possible sequential co-occurrence frequencies to calculate the initial CICs. We then infer the mutation orders with the initial CICs to repredict the sequential co-occurrence frequencies, repeat CIC computation and inference of the mutation orders until a convergent result is obtained.

Based on the principle of maximum entropy, we first use a uniform prior distribution of the occurrence orders, which means that for the non-sequential co-occurrence frequency of the mutation of two genes *i* and *j*, the two mutation orders of *i*→*j* and *j*→*i* occur with the same probability. Therefore, the necessary sequential co-occurrence frequency is set as a half of the corresponding non-sequential frequency. With this setting, we compute the initial CIC between every pair of cancer genes.

We then compute the CIC that an order of more than two mutant genes possesses. In this computation, we must consider that each of the preceding genes may send the carcinogenesis information in parallel to a target gene within the order. Therefore, we borrow the principle of computing resistance in a circuit, which is a parallel-by-serial procedure; we sum all the parallel CICs from the preceding genes to a target gene within the order to determine the *phase CIC* of the order and then formulate the *order CIC* by cascading all the *phase CIC*s. Consider the order *APC→ATM→KRAS* as an example; this order contains two phases of information sending, *→ATM* and *→KRAS*. During the first phase, the information can be sent from only one source, *APC*. Therefore,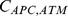, the CIC from *APC* to *ATM*, simply becomes the CIC of the first phase. In the second phase, however, both *APC* and *ATM* can become the information source, requiring the summation of the two parallel CICs as the CIC of the second phase. After the parallel step of each phase, the reciprocals of *phase CIC*s, regarded as resistances, are serially summed as the reciprocal of the *order CIC*. The steps are summarized as follows:

*Parallel step*: 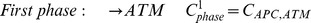


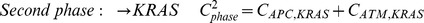

*Series step*: 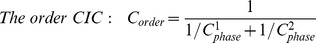.

The *k*th gene in the order is the information receiving gene at the *(k-1)*th phase and has *k-1* senders of parallel information. An order consisting of *n* genes has *n-1* phases of carcinogenesis information conduction. In general, we have the equation, 
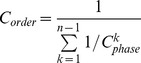
where 
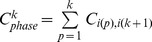
 is the CIC of phase *k*, 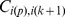 is the CIC from gene 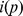 to gene 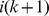, and 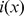 is the index of the gene at position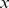 in the order.

Based on the definition of the CIC, a larger CIC value of a possible order implies easier carcinogenesis information conduction within the order. Among all competing orders, the larger the CIC value of an order, the greater probability the occurrence of the order. Therefore, we presume that the CIC of an order is positively proportional to the probability of that order occurring. When estimating the probability of every potential order by a linear mapping from the CICs of all potential orders for a given set of mutant genes, the total of the probabilities of all the potential orders is equal to one. Formally, for a sample with *n* mutant cancer genes, the number of potential orders is *n*!; we map the CIC of order *m* (*m* = 1, 2, …, *n*!) into its probability using the equation 
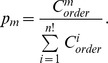



After determine the probabilities of every possible order of the mutations, we redetermine the predicted sequential co-occurrence frequencies as follows: 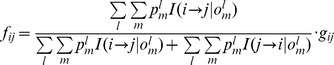
where 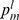 is the probability of order *m* of sample *l*, 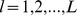, and *L* is the number of samples in question. 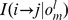 is an indicator function that equals 1 when gene *i* occurs before gene *j* in order *m* of sample *l* and equals 0 in all other cases, and 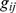 is the non-sequential co-occurrence frequency between gene *i* and gene *j*. If the redetermined 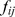 values are nearly identical to the old ones or become convergent, the computed CICs and thus the inferred order probabilities can be regarded as reliable outcomes. Otherwise, the CICs and the order probabilities have to be redetermined in a new loop. The iterative procedure continues in this manner until convergence is reached. In practice, the criterion of convergence can be regarded as satisfied when the absolute difference between the new and old values of 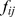 monotonically reduces to a sufficiently small value.

Because we begin the iterative procedure with an initial prediction of the sequential co-occurrence frequencies from non-sequential frequencies based on the maximum entropy principle, which provides the maximum modification potential of the sequential co-occurrence frequencies in the first iteration, the modification will decrease gradually and finally become insignificant. This premise was verified in the study; a satisfying convergence was reached within fewer than 10 loops of the inference procedure using a set of samples from the COSMIC database.

The iteration based on COSMIC data reaches convergence within 10 loops. Here, we use the computation of CIC from *KRAS* to *APC* to introduce the procedure in detail. Initially, we calculate the occurrence frequencies of 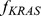 = 125 and 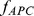 = 209 and a non-sequential co-occurrence frequency 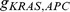 = 79 from the COSMIC database. By defining half of the non-sequential co-occurrence frequency (79) as the sequential frequency, we determine that 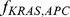 = 39.5. When comparing with the sequential co-occurrence frequencies from genes other than *KRAS* to the gene *APC* in each of the 79 samples, 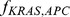 is found to have an average order of 1.47. Therefore the priority 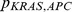 = 1.47, and the initial value of 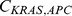 = (39.5/125)*(39.5/209)/1.47^2^ = 0.028.

Using the initial CICs between all cancer gene pairs, we estimate the probability of every potential mutation occurrence order in each sample in the manner described above. According to the probabilities, the non-sequential co-occurrence frequencies can be unevenly divided into sequential frequencies. For the 79 samples in this example, the ratio of *KRAS*→*APC* vs. *APC*→*KRAS* based on the corresponding total probability for each order is 0.28: 0.72. Therefore, we update the value of 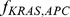 = 79*0.28 = 22.1, and the priority 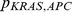 is then determined with the new 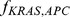. With these new values, we redetermine 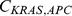.

**Figure 2:**
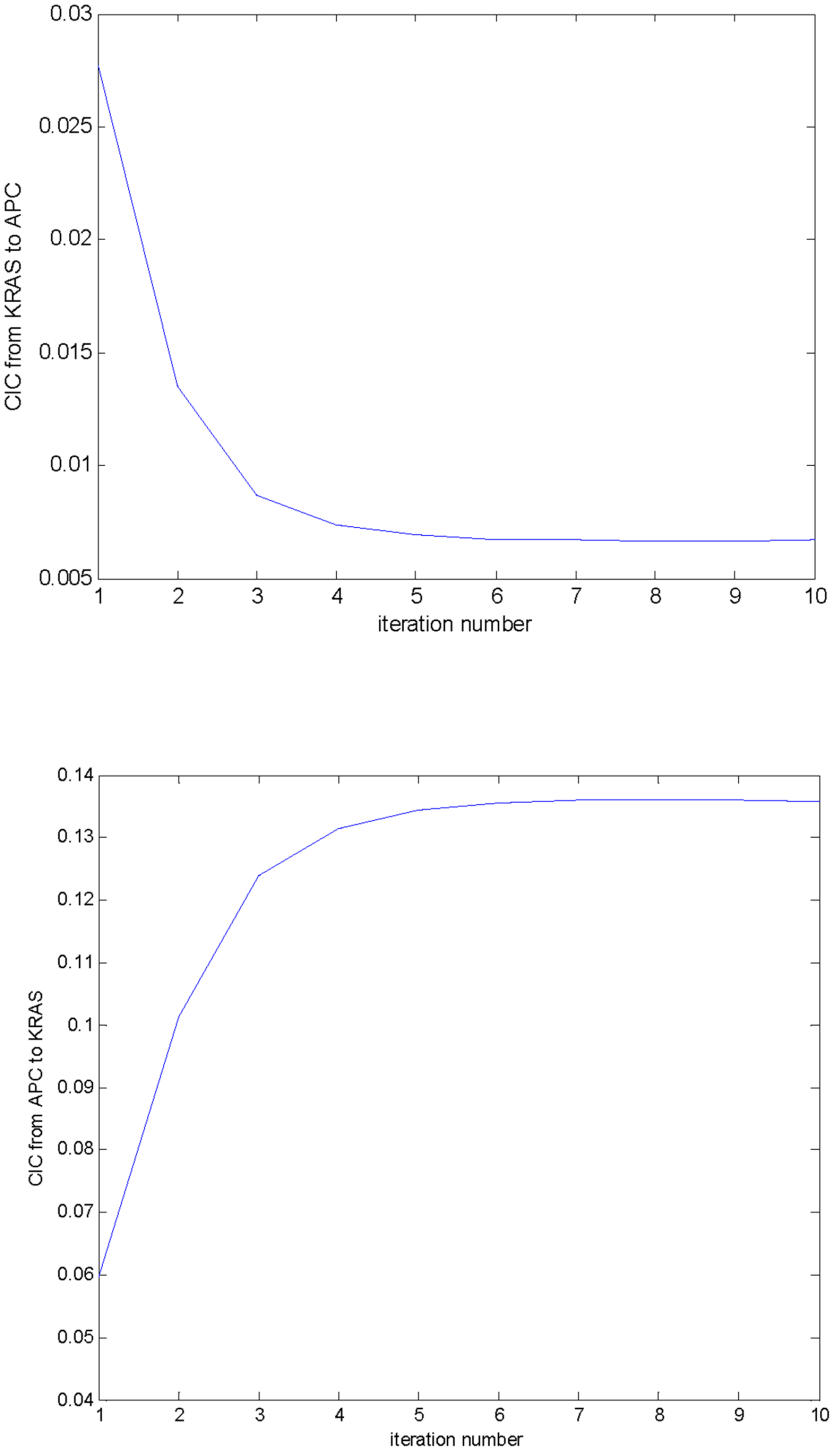
Convergence of iterative computation of CICs. The CICs of 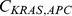 (a) and its counterpart 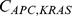 (b) quickly reach convergent as the iterations of computation are performed. After 6 iterations, a satisfying convergence has been reached.

The convergence of 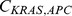 and its counterpart 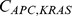 during the iterations is shown in Fig. 2. This example demonstrates that the values reach a satisfying convergence after just 6 iterations. This example also represents the common situation, thus we ended the computation of CICs after 10 iterations in this study.

### Complexity of the inference procedure

CIC computation has a complexity of *O*(*n* ^2^) if the number of cancer genes in the study is *n*, and the inference of the probabilities of all potential orders for a sample with *m* mutant cancer genes has a complexity of *O*(*m!m* ^2^). In our study, *n* is equal to 397 and *m* ranges from 2 to 8. Therefore, the complexity of *O*(*m!m* ^2^) can differ greatly for different samples. In reality, during the inference for the 1,118 samples reported in the study, the majority of the time was consumed by a few samples with the maximum number of mutant cancer genes. It is worth noting that during the entire procedure, we only have to compute the CICs once in each loop to infer the order probabilities for all samples. The inference procedure with 10 iterations for the 1,118 samples was completed within 10 minutes on a platform consisting of a PC (4*2.66 GHz Quad CPU) and Matlab.

### Study data

The results reported in this study were obtained from a recent COSMIC database (issued on September 12^th^, 2012) on coding point mutations. It is a table file containing the names of the mutated cancer genes in each cancer sampled. Mutant genes in the same cancer have the same tumor ID (*ID_tumour*), and the fields of *genome-wide-screen* and *primary side* provide the necessary information used in this study.

### Steps for determining the occurrence and co-occurrence frequencies of cancer gene mutations in the samples

The occurrence and co-occurrence frequencies of cancer genes in the cancer samples were used to estimate the CICs in the study, and the basic statistics were determined using the following steps:

1. Download the *source* file *CosmicMutantExport_v61_120912.tsv* through ftp://ftp.sanger.ac.uk/pub/CGP/cosmic/data_export/;
2. Make a *temporary* file by obtaining the records with the value ‘*y*’ in the ‘*genome-wide screen*’ field from the *source* file;
3. Make a *primary* file by obtaining the records of cancer genes defined by the file *Table_1_full_2012-03-15.xls* in the *Cosmic* web site from the *temporary* file, and refining the records into sequences of *Gene_name* and *ID_Sample*;
4. Make a *mutation_sequence* file in which each record is a list of the mutated genes in the same sample based on the *primary* file, and discard the record that contains only one gene name in the *mutation_sequence* file;
5. Count the occurrence and co-occurrence frequencies of the cancer genes based on the *mutation_sequence* file.

## Results

### Features of the estimated CICs

**Figure 3:**
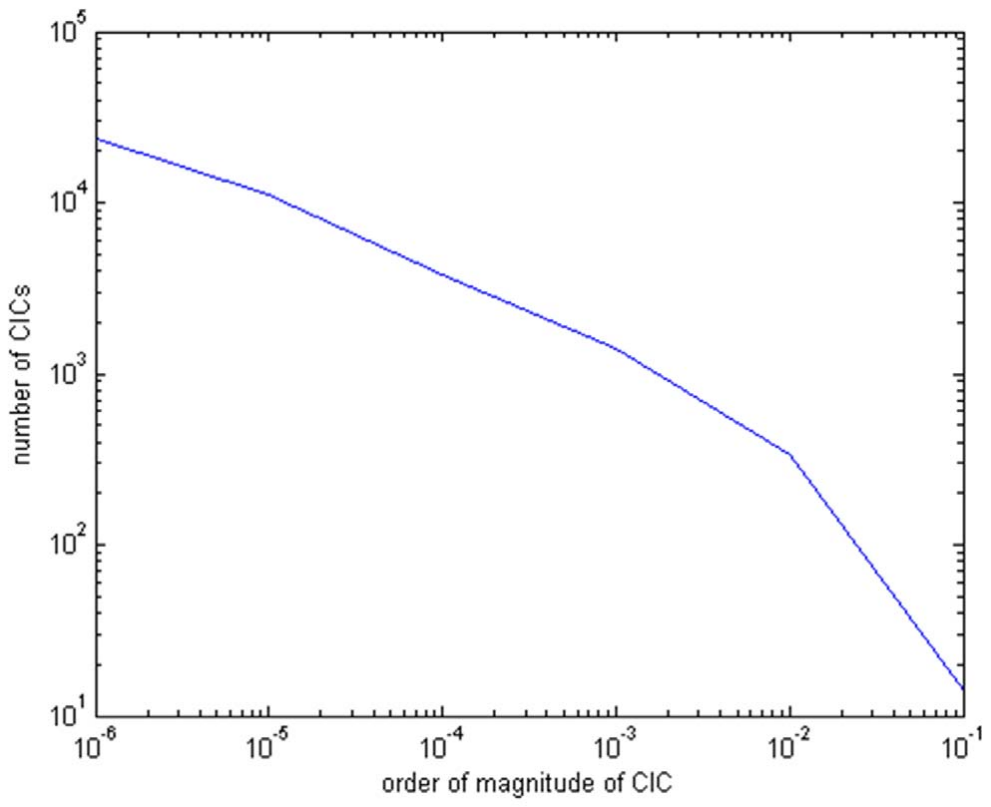
The power law-like distribution of CICs. CICs greater than 1.0E-6 are present in a power law-like distribution; specifically, the logarithm of the number of the CICs versus the logarithm of their orders of magnitude yields a piecewise linear relationship. Based on the question of whether a power law distribution is appropriate for analyzing complex networks and worries about the unreliability of undervalued CICs that might be caused by spare data, only the CICs greater than 1.0E-6 were directly used in the inference in this study. CICs inferred as less than 1.0E-6 were replaced by the threshold for smoothing.

**Figure 4:**
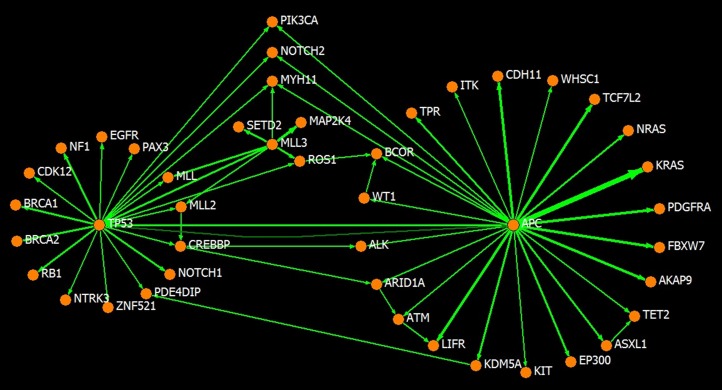
An illustration of the asymmetry of CIC-linked cancer gene networks. Forty-four frequently mutated cancer genes (in more than 20 genome-wide scanned samples in the COSMIC database) are illustrated with the CICs between them larger than 1.0E-2. The thickness of the link is proportional to the strength of the corresponding CIC. When a pair of genes has bidirectional links, the stronger link is drawn as a straight line and the weaker one is drawn as a curved line (see the case of *APC* ←→*TP53*). The asymmetry can be observed by the fact that no bidirectional links of similar strengths exist between gene pairs, and *APC*, *TP53*, and *MLL3* each play a hub role in the network.

We performed the inference on cancer gene mutation data from genome-wide scanned samples collected in a recent version of the COSMIC database. A total of 1,212 samples harboring 6,281 mutations in 397 cancer genes was available for determining the basic occurrence and co-occurrence frequencies. From these, 1,118 samples, each harboring no more than 8 mutant cancer genes, were used in the iterative procedure of CIC computation and order inference. Table S1 lists the 1,118 samples. The results were found to converge within 10 iterations. After convergence, CICs with a value greater than 1.0E-6 presented a power law-like distribution over the magnitudes, such that the overwhelming majority has a magnitude less than the average of 4.0E-4 and a very small portion has a larger than average magnitude (Fig. 3, Table S2). This feature is also true for the distribution of the magnitudes of the CICs from (or to) a given gene in most cases, which means that only a small number of partners are significant in terms of carcinogenesis information conduction for any given gene. In other words, the CICs identify the closest partners in carcinogenesis information conduction. Furthermore, the directed networks of cancer genes linked by the CICs were asymmetrical and small world-like. The CIC from gene *i* to gene *j* was usually unequal to that from gene *j* to gene *i*; the network has a number of hub genes with many more links than normal. This feature is consistent with the notion that the signaling network in cancer is analogous to the Internet, which constructs a small world with hub nodes [27]–[29]. Fig. 4 illustrates a CIC linked network covering 44 cancer genes, including the hub genes *APC*, *TP53* and *MLL3*, and the links stronger than 1.0E-2 showing asymmetry. The asymmetry of the CICs implies the existence of a preference for certain mutation orders. Additionally, the three hub genes are all tumor-suppressor genes, and the strongest directed link, with a value of 0.136, is from *APC* to *KRAS*, one of the most frequently mutated oncogenes, suggesting a superior information channel from the mutation of *APC* to the mutation of *KRAS*.

### The inference of probable orders

The inferred mutation orders with a probability higher than random chance, referred to hereafter as *probable orders*, provided more concrete insights into tumor progression. We analyzed the probable orders inferred for the 1,118 cancer samples in question to investigate a maximum of 8 mutation steps from initiation. The primary sites of the samples were mainly located in the *ovary* (256), *large_intestine* (*LI*, 180), *haematopoietic_and_lymphoid_tissue* (*HLT*, 148), *prostate* (100), *breast* (97), *central_nervous_system* (*CNS*, 86), and *upper_aerodigestive_tract* (*UAT*, 72).

Table S3 lists all the probable orders and their probabilities in the analyzed samples, and Table 1 shows a selection of them. Based on the probable orders, we concluded that in a given sample only a small portion of all the potential orders has a probability higher than random chance, and the sum total of the probabilities of those orders is close to the number of samples with a ratio of 1034.4/1118. This indicates that the inference identified a small portion of all the potential orders permutated by the given set of mutant cancer genes as the probable orders. For a sample harboring two mutant cancer genes, the inference always strongly suggests one of the two potential orders. However, for the samples with more than two mutant cancer genes, some orders might have comparable high probabilities. Although we cannot judge the individual plausibilities of the inferred probable orders because of the lack of ground truth for the orders in most cases, their significance could be strongly suggested by evaluating the inference with samples of a certain cancer type that have been well studied in terms of order. For example, *APC, KRAS and TP53* are the three most frequently mutated genes in colon cancers, and their mutation orders have been well modeled [30], [31]. In our results, the sample with mutant cancer genes *APC* and *KRAS*, yielded an inferred probability of 0.95 for the order *APC*→*KRAS*, which was consistent with previous studies. For the sample with mutant *APC, KRAS and TP53* cancer genes, three probable orders of *APC→KRAS→TP53* (0.33), *APC→TP53→KRAS* (0.32) *and TP53→APC→KRAS* (0.19) were inferred from 6 potential ones, and this result was also consistent with previous studies. *BRCA1* germline mutations confer a high risk of breast and ovarian cancer, but somatic loss of the wild-type *BRCA1* allele has been shown to usually occur *after* mutation of *TP53* [32]. In agreement with this observation, we inferred the somatic mutation order *TP53→BRCA1* with a probability greater than 0.99. These examples provide evidence to support the inference validity.

**Table 1:** Examples of cancer gene mutation orders with a predicted probability greater than random chance.

Order	Probability	Order	Probability
*APC→BRCA2*	*0.99932*	*APC→ATM→KRAS*	*0.39011*
*APC→FBXW7*	*0.99675*	*APC→KRAS→ATM*	*0.41748*
*APC→KRAS*	*0.95281*	*APC→BRAF→TP53*	*0.20884*
*APC→NOTCH2*	*0.99906*	*APC→TP53→BRAF*	*0.34198*
*APC→PTEN*	*0.99986*	*TP53→APC→BRAF*	*0.2958*
*APC→SMARCA4*	*0.99934*	*APC→KRAS→TP53*	*0.33317*
*APC→TP53*	*0.65624*	*APC→TP53→KRAS*	*0.32532*
*ARID1A→ATM*	*0.99991*	*TP53→APC→KRAS*	*0.18769*
*ARID1A→CTNNB1*	*0.99987*	*APC→MLL3→PTEN*	*0.49936*
*ARID1A→PTEN*	*0.99966*	*APC→PTEN→MLL3*	*0.49797*
*ARID2→CTNNB1*	*0.99992*	*CREBBP→KRAS→ARID1A*	*0.36621*
*BRAF→FBXW7*	*0.99994*	*CREBBP→ARID1A→KRAS*	*0.56109*
*BRAF→PTEN*	*0.99792*	*CREBBP→CTNNB1→SMARCA4*	*0.27984*
*CREBBP→BRCA2*	*0.9995*	*CREBBP→SMARCA4→CTNNB1*	*0.71849*
*CREBBP→CTNNB1*	*0.99906*	*MLL3→SMARCA4→EP300*	*0.49603*
*CTNNB1→SMARCA4*	*0.7542*	*MLL3→EP300→SMARCA4*	*0.4967*
*EZH2→CARD11*	*0.99922*	*PIK3CA→SMARCA4→CTNNB1*	*0.46455*
*EZH2→CTNNB1*	*0.96595*	*PIK3CA→CTNNB1→SMARCA4*	*0.52713*
*KRAS→FBXW7*	*0.75719*	*TP53→MLL3→ARID1A*	*0.48265*
*KRAS→PTEN*	*0.99874*	*TP53→ARID1A→MLL3*	*0.45573*
*MLL2→CTNNB1*	*0.99989*	*TP53→MLL3→ARID2*	*0.4851*
*MYH11→SMARCA4*	*0.99606*	*TP53→ARID2→MLL3*	*0.46217*
*NOTCH2→ARID1A*	*0.99964*	*TP53→CTNNB1→ATM*	*0.49888*
*NRAS→SMARCA4*	*0.95881*	*TP53→ATM→CTNNB1*	*0.50012*
*PIK3CA→ARID1A*	*0.99971*	*TP53→ROS1→BRAF*	*0.73047*
*PIK3CA→EP300*	*0.99954*	*TP53→BRAF→ROS1*	*0.26871*
*TP53→ARID1A*	*0.99985*	*TP53→PIK3CA→BRCA2*	*0.49745*
*TP53→AKAP9*	*0.99941*	*TP53→BRCA2→PIK3CA*	*0.49757*
*TP53→ARID2*	*0.99985*	*TP53→EZH2→CREBBP*	*0.47725*
*TP53→ATM*	*0.99974*	*TP53→CREBBP→EZH2*	*0.52201*
*TP53→BRAF*	*0.99937*	*TP53→MLL2→CREBBP*	*0.56795*
*TP53→BRCA2*	*0.99827*	*TP53→CREBBP→MLL2*	*0.43152*
*TP53→CARD11*	*0.99926*	*TP53→NOTCH2→CREBBP*	*0.50977*
*TP53→CREBBP*	*0.99989*	*TP53→CREBBP→NOTCH2*	*0.4884*
*TP53→CTNNB1*	*0.99984*	*TP53→FBXW7→CTNNB1*	*0.47758*
*TP53→EP300*	*0.99894*	*TP53→CTNNB1→FBXW7*	*0.47344*
*TP53→EZH2*	*0.99985*	*TP53→KRAS→CTNNB1*	*0.42094*
*TP53→KRAS*	*0.83612*	*TP53→CTNNB1→KRAS*	*0.4181*
*TP53→MLL*	*0.99992*	*TP53→MLL2→EZH2*	*0.5875*
*TP53→MLL2*	*0.9999*	*TP53→EZH2→MLL2*	*0.41196*
*TP53→MLL3*	*0.98377*	*TP53→KRAS→FBXW7*	*0.38181*
*TP53→MYH11*	*0.99954*	*TP53→FBXW7→KRAS*	*0.31798*
*TP53→NRAS*	*0.94242*	*TP53→PIK3CA→KRAS*	*0.40396*
*TP53→PIK3CA*	*0.99789*	*TP53→KRAS→PIK3CA*	*0.36917*
*TP53→PTEN*	*0.99989*	*TP53→PIK3CA→MYH11*	*0.49797*
*TP53→SMARCA4*	*0.99988*	*TP53→MYH11→PIK3CA*	*0.49427*

The examples in the table are selected based on that the genes have a mutant frequency greater than 40 in the COSMIC database to show the estimation for the common cases. Due to the limited space, the prediction for the samples with more than 3 mutant cancer genes is not shown. Refer to Table S3 for a complete result.

The random chance is 1/*n!*, where *n* is the number of mutant cancer genes in the sample.

Note, any two samples with the same set of mutant genes have identical predicted results.

### Initiators of probable mutation orders

Identifying the initiators of mutation orders has been regarded as one of the major challenges in the study of tumor progression [1]. Our inferred probable orders of mutation provided informative hints to solving this challenge. By examining the genes that initiate the probable orders, we found that the initiators were dominated by tumor-suppressor genes. An overwhelming majority (more than 77.5%) of the probability-weighted number of the probable orders was inferred to be initiated by a tumor-suppressor gene rather than an oncogene. There were 368 cancer genes in the test cancer samples, among them only 92 were tumor suppressors. More specifically, there were 1,858 mutations of tumor suppressors among totally 3,823 mutations of all the cancer genes. Therefore the average chance for tumor suppressors to initiate the mutation orders was 48.6% (1858/3823). This demonstrates that the dominance of tumor suppressors in initiating the mutation orders could not be ascribed to chance. Additionally, the ratios of the number of times a gene was the initiator to its mutation frequency were generally different, implying that it is not certain that frequently mutated genes will mutate early (Table 2). Significantly, the probability-weighted number of the probable orders started by the top two tumor-suppressor gene initiators *TP53* and *APC*, consisted of percentages as large as 46.9% and 11.4%, respectively. In contrast, the top two oncogene initiators, *PIK3CA* and *KRAS*, were found in percentages as small as 3.1% and 1.3%, respectively. The top initiators of mutation at the respective primary cancer sites suggested more details (Table 3). In general, all cancers at the major primary sites of the samples revealed a tumor-suppressor gene as their top initiator. In particular, *TP53* was a common top initiator in four of the previously listed cancer types, *ovary*, *UAT*, *breast* and *prostate*, with percentages of 91.5%, 73.4%, 57.6% and 30.4%, respectively. In *LI* cancers, the top initiator was *APC* (57.5%), followed by *TP53* (29.7%). Both *CNS* and *HLT* cancers had no obviously superior initiators, with *CIC* (13.6%), *PIK3CA* (10.1%) and *TP53* (10.0%) as the top three initiators for the former, and *TP53* (14.9%), *NPM1* (10.4%) and *MLL2* (9.9%) as the top three initiators for the latter. From the perspective of initiator distribution, *ovary*, *LI*, *UAT* and *breast* cancers were inferred to be dominated by a small number of tumor-suppressor genes, while *HLT*, *CNS* and *prostate* cancers were inferred to have more diverse significant initiators.

**Table 2:** The type of a cancer gene and its probability-weighted times of starting an order with a probability greater than random chance.

Gene	Type	Times	Rate	Gene	Type	Times	Rate
*TP53*	*s*	*484.732*	*0.844*	*XPO1*	*o*	*1*	*0.077*
*APC*	*s*	*117.471*	*0.675*	*TIF1*	*o*	*1*	*0.125*
*PIK3CA*	*o*	*31.97*	*0.381*	*ERCC5*	*s*	*1*	*0.083*
*MLL3*	*s*	*26.927*	*0.481*	*ELN*	*o*	*1*	*0.125*
*KRAS*	*o*	*24.093*	*0.223*	*LRIG3*	*o*	*1*	*0.167*
*MLL2*	*s*	*20.235*	*0.355*	*LMO1*	*o*	*1*	*0.5*
*CREBBP*	*s/o*	*17.294*	*0.402*	*ASPSCR1*	*o*	*1*	*1*
*ATM*	*s*	*14.404*	*0.4*	*NCOA1*	*o*	*1*	*0.143*
*ARID1A*	*s*	*13.625*	*0.296*	*SRSF2*	*o*	*1*	*0.5*
*NPM1*	*s*	*12.96*	*0.48*	*CBL*	*s/o*	*1*	*0.2*
*EZH2*	*s*	*10.733*	*0.335*	*MYD88*	*o*	*1*	*0.125*
*CIC*	*s*	*10.649*	*0.41*	*NUP98*	*o*	*1*	*0.01*
*ARID2*	*s*	*8.699*	*0.335*	*PTPN11*	*o*	*1*	*0.2*
*ROS1*	*o*	*7.888*	*0.316*	*CDH1*	*s*	*0.999*	*0.062*
*TET2*	*s*	*7.049*	*0.441*	*PER1*	*o*	*0.999*	*0.125*
*WT1*	*s*	*7.027*	*0.502*	*MEN1*	*s*	*0.999*	*0.1*
*CTNNB1*	*o*	*6.862*	*0.114*	*PRDM1*	*s*	*0.999*	*0.125*
*PBRM1*	*s*	*6.854*	*0.685*	*IL21R*	*o*	*0.998*	*0.25*
*PTEN*	*s*	*6.801*	*0.235*	*MUC1*	*o*	*0.997*	*0.332*
*BRAF*	*o*	*5.988*	*0.23*	*PIK3R1*	*s*	*0.997*	*0.066*
*NOTCH2*	*o*	*5.907*	*0.281*	*TRIP11*	*o*	*0.996*	*0.1*
*MYST4*	*o*	*5.492*	*0.25*	*ITK*	*o*	*0.995*	*0.059*
*SMARCA4*	*s*	*5.45*	*0.156*	*NCOA2*	*o*	*0.995*	*0.199*
*DNMT3A*	*s*	*5.293*	*0.23*	*BCOR*	*s*	*0.995*	*0.059*
*ASXL1*	*s*	*5.064*	*0.362*	*TSHR*	*o*	*0.995*	*0.124*
*MYH9*	*o*	*4.988*	*0.416*	*MAP2K4*	*s*	*0.994*	*0.076*
*KDM5A*	*o*	*4.987*	*0.623*	*GNAS*	*o*	*0.994*	*0.062*
*NSD1*	*o*	*4.965*	*0.414*	*MSH2*	*s*	*0.993*	*0.199*
*MYH11*	*o*	*4.561*	*0.198 = = = *	*SRGAP3*	*o*	*0.993*	*0.165*
*BCL2*	*o*	*4.165*	*0.116*	*FGFR2*	*o*	*0.992*	*0.142*
*RET*	*o*	*3.991*	*0.333*	*FANCD2*	*s*	*0.991*	*0.33*
*EP300*	*s*	*3.991*	*0.174*	*MET*	*o*	*0.991*	*0.099*
*ALK*	*o*	*3.972*	*0.306*	*EWSR1*	*o*	*0.991*	*0.142*
*PHF6*	*s*	*3.264*	*0.297*	*BRIP1*	*s*	*0.99*	*0.165*
*BLM*	*s*	*3.076*	*0.22*	*SETD2*	*s*	*0.99*	*0.062*
*IDH2*	*o*	*3*	*0.214*	*BCL6*	*o*	*0.989*	*0.11*
*COL1A1*	*o*	*2.997*	*0.231*	*WHSC1*	*o*	*0.985*	*0.123*
*GNA11*	*o*	*2.996*	*0.749*	*BAP1*	*s*	*0.983*	*0.089*
*SF3B1*	*o*	*2.993*	*0.136*	*ETV6*	*o*	*0.953*	*0.136*
*TPR*	*o*	*2.987*	*0.373*	*FLI1*	*o*	*0.902*	*0.18*
*CDH11*	*o*	*2.987*	*0.187*	*DNM2*	*s*	*0.89*	*0.064*
*PDGFRA*	*o*	*2.971*	*0.186*	*BCR*	*o*	*0.888*	*0.296*
*MLL*	*o*	*2.847*	*0.142*	*VHL*	*s*	*0.818*	*0.055*
*KIT*	*o*	*2.59*	*0.173*	*FANCC*	*s*	*0.711*	*0.711*
*MED12*	*o*	*2.514*	*0.168*	*MAF*	*o*	*0.682*	*0.341*
*FBXW7*	*s*	*2.428*	*0.069*	*RUNX1*	*o*	*0.65*	*0.043*
*RB1*	*s*	*2.204*	*0.092*	*CARD11*	*o*	*0.5*	*0.019*
*LIFR*	*o*	*2.175*	*0.128*	*LHFP*	*o*	*0.5*	*0.167*
*AKAP9*	*o*	*2.156*	*0.108*	*NUMA1*	*o*	*0.453*	*0.032*
*NRAS*	*o*	*2.037*	*0.06*	*SUZ12*	*o*	*0.397*	*0.066*
*EGFR*	*o*	*2*	*0.154*	*KIAA1549*	*o*	*0.319*	*0.106*
*JAK2*	*o*	*2*	*0.2*	*NCOA4*	*o*	*0.314*	*0.157*
*PALB2*	*s*	*1.991*	*0.153*	*FHIT*	*o*	*0.257*	*0.086*
*NF1*	*s*	*1.989*	*0.071*	*TNFAIP3*	*s*	*0.254*	*0.023*
*JAK1*	*o*	*1.986*	*0.397*	*NFKB2*	*o*	*0.22*	*0.11*
*PRDM16*	*o*	*1.97*	*0.246*	*MDM4*	*o*	*0.198*	*0.066*
*CAMTA1*	*o*	*1.703*	*0.122*	*H3F3A*	*o*	*0.197*	*0.197*
*NOTCH1*	*o*	*1.681*	*0.06*	*HIP1*	*o*	*0.196*	*0.028*
*CCND2*	*o*	*1.628*		*SUFU*	*s*	*0.188*	*0.047*
*SMARCB1*	*s*	*1.601*	*0.543*	*SBDS*	*s*	*0.184*	*0.092*
*NF2*	*s*	*1.5*	*0.133*	*JAK3*	*o*	*0.144*	*0.013*
*FOXP1*	*o*	*1.5*	*0.3*	*HOOK3*	*o*	*0.11*	*0.026*
*CD74*	*o*	*1.499*	*0.15*	*TCF7L2*	*o*	*0.094*	*0.006*
*CCND3*	*o*	*1.404*	*0.75*	*EIF4A2*	*o*	*0.069*	*0.007*
*PIM1*	*o*	*1.402*	*0.14*	*ERBB2*	*o*	*0.06*	*0.003*
*SLC45A3*	*o*	*1.384*	*0.175*	*ZNF331*	*o*	*0.052*	*0.013*
*IL7R*	*o*	*1.27*	*0.154*	*C15orf55*	*o*	*0.046*	*0.011*
*BTG1*	*o*	*1.174*	*0.159*	*HOXA11*	*o*	*0.045*	*0.015*
*ZNF521*	*o*	*1.148*	*0.196*	*PHOX2B*	*s*	*0.018*	*0.006*
*PDE4DIP*	*o*	*1.099*	*0.057*	*PCM1*	*o*	*0.008*	*0.002*
*MSH6*	*s*	*1.017*	*0.061*	*MALT1*	*o*	*0.003*	*0.001*
*LMO2*	*o*	*1*	*0.17*	**Total**		*1034.4*	

*s*: tumor-suppressor gene, *o*: oncogene. The ratios of the summed frequencies of *s*, *o*, and *s/o* to the total are 0.775, 0.207 and 0.018, respectively.

Rate is the ratio of Times to the gene's total mutation number in the samples in question.

**Table 3:** The top initiators of the probable mutation orders in different cancer types.

Cancer	Gene	Type	Percent	Cancer	Gene	Type	Percent
Ovary	*TP53*	*s*	*91.5*	Prostate	*NCOA1*	*o*	*1.1*
Ovary	*APC*	*s*	*1.7*	Prostate	Others		*18.9*
Ovary	*PIK3CA*	*o*	*1.5*	Prostate	**Total**	*s*	***59.0***
Ovary	*ARID1A*	*s*	*0.8*	Prostate	**Total**	*o*	***39.9***
Ovary	*KRAS*	*o*	*0.7*	Breast	*TP53*	*s*	*57.6*
Ovary	*NF2*	*s*	*0.4*	Breast	*PIK3CA*	*o*	*11.8*
Ovary	*BRAF*	*o*	*0.4*	Breast	*MLL3*	*s*	*4.7*
Ovary	*RB1*	*s*	*0.4*	Breast	*PTEN*	*s*	*2.2*
Ovary	*MYST4*	*o*	*0.4*	Breast	*ATM*	*s*	*2.1*
Ovary	*GNA11*	*o*	*0.4*	Breast	*ROS1*	*o*	*2.1*
Ovary	*EGFR*	*o*	*0.4*	Breast	*AKAP9*	*o*	*1.1*
Ovary	*LIFR*	*o*	*0.4*	Breast	*PRDM16*	*o*	*1.1*
Ovary	*MYH9*	*o*	*0.4*	Breast	*SMARCA4*	*s*	*1.1*
Ovary	*KIT*	*o*	*0.4*	Breast	*MYH11*	*o*	*1.1*
Ovary	*MLL3*	*s*	*0.3*	Breast	*MYH9*	*o*	*1.1*
Ovary	**Total**	*s*	***95.1***	Breast	*ASPSCR1*	*o*	*1.1*
Ovary	**Total**	*o*	***4.9***	Breast	*ARID1A*	*s*	*1.1*
LI	*APC*	*s*	*57.5*	Breast	*PDGFRA*	*o*	*1.1*
LI	*TP53*	*s*	*29.7*	Breast	*BRAF*	*o*	*1.1*
LI	*KRAS*	*o*	*6.8*	Breast	*NSD1*	*o*	*1.1*
LI	*ATM*	*s*	*1.2*	Breast	*PDE4DIP*	*o*	*1.1*
LI	*PIK3CA*	*o*	*0.9*	Breast	*MAP2K4*	*s*	*1.1*
LI	*NSD1*	*o*	*0.6*	Breast	*FANCD2*	*s*	*1.1*
LI	*IL21R*	*o*	*0.6*	Breast	*MET*	*o*	*1.1*
LI	*GNA11*	*o*	*0.6*	Breast	Others		*4.1*
LI	*ALK*	*o*	*0.6*	Breast	**Total**	*s*	***75.2***
LI	*PDGFRA*	*o*	*0.6*	Breast	**Total**	*o*	***24.7***
LI	*ARID1A*	*s*	*0.5*	CNS	*CIC*	*s*	*13.6*
LI	*MYH11*	*o*	*0.3*	CNS	*PIK3CA*	*o*	*10.1*
LI	*TCF7L2*	*o*	*0.1*	CNS	*TP53*	*s*	*10*
LI	**Total**	*s*	***88.9***	CNS	*MLL3*	*s*	*5.7*
LI	**Total**	*o*	***11.0***	CNS	*MLL2*	*s*	*5.5*
HLT	*TP53*	*s*	*14.9*	CNS	*CTNNB1*	*o*	*5.2*
HLT	*NPM1*	*s*	*10.4*	CNS	*CREBBP*	*s/o*	*5.1*
HLT	*MLL2*	*s*	*9.9*	CNS	*ATM*	*s*	*5.1*
HLT	*EZH2*	*s*	*7.6*	CNS	*SMARCA4*	*s*	*4.4*
HLT	*WT1*	*s*	*5.6*	CNS	*NOTCH2*	*o*	*3.7*
HLT	*TET2*	*s*	*4.8*	CNS	*APC*	*s*	*2.5*
HLT	*DNMT3A*	*s*	*4.2*	CNS	*KDM5A*	*o*	*2.5*
HLT	*BCL2*	*o*	*3.3*	CNS	*ROS1*	*o*	*2.5*
HLT	*PHF6*	*s*	*2.6*	CNS	*AKAP9*	*o*	*1.4*
HLT	*IDH2*	*o*	*2.4*	CNS	*COL1A1*	*o*	*1.3*
HLT	*RET*	*o*	*2.4*	CNS	*TIF1*	*o*	*1.3*
HLT	*ASXL1*	*s*	*2.4*	CNS	*ARID2*	*s*	*1.3*
HLT	*CREBBP*	*s/o*	*2.1*	CNS	*TPR*	*o*	*1.3*
HLT	*NRAS*	*o*	*1.6*	CNS	*EP300*	*s*	*1.3*
HLT	*ATM*	*s*	*1.6*	CNS	*BLM*	*s*	*1.3*
HLT	*CDH11*	*o*	*1.6*	CNS	Others		*14.9*
HLT	*KRAS*	*o*	*1.6*	CNS	**Total**	*s*	***56.6***
HLT	*CD74*	*o*	*1.2*	CNS	**Total**	*o*	***38.3***
HLT	*CCND3*	*o*	*1.1*	UAT	*TP53*	*s*	*73.4*
HLT	*PIM1*	*o*	*1.1*	UAT	*NSD1*	*o*	*3*
HLT	Others		*17.6*	UAT	*NOTCH1*	*o*	*2.4*
HLT	**Total**	*s*	***67.5***	UAT	*FBXW7*	*s*	*1.9*
HLT	**Total**	*o*	***29.6***	UAT	*MLL3*	*s*	*1.9*
Prostate	*TP53*	*s*	*30.4*	UAT	*CREBBP*	*s/o*	*1.5*
Prostate	*APC*	*s*	*9.9*	UAT	*EP300*	*s*	*1.5*
Prostate	*MLL3*	*s*	*4.5*	UAT	*FOXP1*	*o*	*1.5*
Prostate	*PTEN*	*s*	*4.3*	UAT	*MED12*	*o*	*1.5*
Prostate	*MYST4*	*o*	*4*	UAT	*GNAS*	*o*	*1.5*
Prostate	*ATM*	*s*	*3.9*	UAT	*JAK1*	*o*	*1.5*
Prostate	*KDM5A*	*o*	*3.3*	UAT	*ASXL1*	*s*	*1.4*
Prostate	*PIK3CA*	*o*	*3.3*	UAT	*MLL2*	*s*	*1.1*
Prostate	*MYH9*	*o*	*2.2*	UAT	*FANCC*	*s*	*1.1*
Prostate	*TPR*	*o*	*2.2*	UAT	*CAMTA1*	*o*	*1.1*
Prostate	*MLL*	*o*	*2*	UAT	*APC*	*s*	*1*
Prostate	*MED12*	*o*	*1.7*	UAT	*CCND2*	*o*	*1*
Prostate	*SLC45A3*	*o*	*1.5*	UAT	*NUMA1*	*o*	*0.5*
Prostate	*RB1*	*s*	*1.3*	UAT	*KIAA1549*	*o*	*0.5*
Prostate	*CREBBP*	*s/o*	*1.1*	UAT	*EZH2*	*s*	*0.4*
Prostate	*PALB2*	*s*	*1.1*	UAT	Others		*0.3*
Prostate	*ZNF521*	*o*	*1.1*	UAT	**Total**	*s*	***83.9***
Prostate	*EGFR*	*o*	*1.1*	UAT	**Total**	*o*	***14.6***
Prostate	*LRIG3*	*o*	*1.1*				

Percent: the sum of the probabilities of the orders initiated by the gene versus the total of the probabilities of the orders in the same cancer type.

At most top 20 initiators are listed for each cancer type due to the limited space.

Previous studies have suggested a number of hallmark functions that need to be acquired for a cancer to generate, helping researchers understand the complexity in tumor progression in a way of logical, scientific manner [33], [34]. Our inferred results point to a suggestion that goes one step further. In most cancers, the earliest acquired hallmark function might be *evading apoptosis* because the majority of first mutated genes in every cancer type in Table 3 (*TP53, APC, KRAS, PIK3CA, NPM1* and *CIC*) have been found to encode apoptosis-regulating proteins, and the mutation of all of these genes has been shown to lead to deficient apoptosis functions. Specifically, the mutation of *TP53* can result in the removal of a key component of the DNA damage sensor, which functions to induce apoptosis [33], [34], mutant forms of the APC protein can attenuate responses to apoptotic stimuli [35], [36], the mutations in *KRAS* and *PIK3CA* can activate pathways that transmit anti-apoptotic survival signals [33], and the proteins encoded by *NPM1* and *CIC* have been shown to function in apoptosis [37], [38].

### Informative transitions in the probable orders

The transitions in the probable orders provided additional information on tumor progression. Though mutations in *BRCA1* and *BRCA2* have been regarded as key markers for breast cancer occurrence, somatic mutations in the two genes in the breast cancer samples were not very frequent, with rates of 3/97 and 6/97, respectively, and both genes were inferred to have no chance of initiating a probable order. However, among all transitions in the probable orders of the breast cancers, *TP53→BRCA2* and *TP53→BRCA1* were identified as the second and fourth most frequent transitions, respectively, implying that mutations in these two genes tend to occur next to the mutation of *TP53*. Similarly, the transition of *TP53→BRCA1*was ranked as the third most frequent in the probable orders in ovarian cancer, supporting the conjecture mentioned above. In *LI* cancers, mutations in *APC*, *TP53* and *KRAS* were found to occur at extraordinarily frequencies with rates of 146/180, 111/180 and 79/180, respectively, and their mutual transitions were the top six most frequent, implying that these three genes as a group play dominating roles in *LI* cancers. Liquid *HLT* cancers were inferred to have the 3 most frequent transitions that converged on one gene, *TP53→BCL2*, *MLL2→BCL2* and *EZH2→BCL2*. Given that *BCL2* is a key anti-apoptotic gene [39] and was the most frequently mutated gene in *HLT* cancer samples, these convergent transitions suggest that *HLT* cancers might acquire the function of evading apoptosis in a unique way, mutation of the key anti-apoptotic gene *BCL2* next to the mutations of certain tumor-suppressor genes. Informatively, among all the 36 *BCL2* mutant samples, mutations at 179C and 392C of CDS (Coding DNA Sequence) were as frequent as 5 and 4 times, respectively, suggesting those to be hotspot mutations that might play a particular role in evading apoptosis. Because *TP53*, *MLL2* and *EZH2* were inferred to be the top initiators of mutation in *HLT* cancer samples, the function of evading apoptosis could be acquired in an early stage of tumor progression.

## Discussion

The inferred results from individual samples firmly revealed the order heterogeneity in a given cancer type, showing the complexity of the disease. The results also highlighted the limited number of genes that are able to initiate the mutations and revealed that the hallmark function of evading apoptosis is acquired early. Other regularities implied in the results might also be significant in understanding and treating the disease.

The proposed approach for inferring the temporal order of mutations is superior to existing methods in two ways: 1) it can be used to infer the order of mutation in individual samples with mutations in multiple genes which have been identified at the same time; and 2) it is completely data-driven, free from the difficulty in existing methods of setting proper parameters, such as *fitness*, *mutation rate* or *waiting time* [9]–[11]. When this approach is better supported by more sufficient data, it is expected to help discover more reliable information to understand the mechanism of carcinogenesis. Fortunately, the wide application of a new generation of sequencing will make this hope a reality.

The key to the success of this study is finding the statistical measurement of CIC, which proved to usually be asymmetrical between a pair of cancer genes, laying the foundation of mutation order inference. Meanwhile, the iterative procedure provides a feasible way to infer the CICs from non-sequential co-occurrence frequencies. With the CICs, the linkages between cancer gene mutations are modeled as a complex network with directed links. The small world-like nature of the complex network makes the inference of the temporal order of mutations effective.

## Supporting Information

Table S1 **Tumor samples used in the inference of orders (xlsx).**
(XLSX)

Table S2 **CICs between cancer genes (xlsx).**
(XLSX)

Table S3 **Orders of mutations in cancer genes with an estimated probability greater than random chance for all 1,118 samples in the inference (xlsx).**
(ZIP)
