# Correction: Inferring the Temporal Order of Cancer Gene Mutations in Individual Tumor Samples

**DOI:** 10.1371/journal.pone.0098676

**Published:** 2014-05-27

**Authors:** The PLOS ONE Staff

There are errors in Table 2. Some numbers in the fourth column (Rate) are misplaced. The authors have provided a corrected table below.

**Table 2:** The type of a cancer gene and its probability-weighted times of starting an order with a probability greater than random chance.

Gene	Type	Times	Rate	Gene	Type	Times	Rate
*TP53*	*s*	*484.732*	*0.844*	*XPO1*	*o*	*1*	*0.077*
*APC*	*s*	*117.471*	*0.675*	*TIF1*	*o*	*1*	*0.125*
*PIK3CA*	*o*	*31.97*	*0.381*	*ERCC5*	*s*	*1*	*0.083*
*MLL3*	*s*	*26.927*	*0.481*	*ELN*	*o*	*1*	*0.125*
*KRAS*	*o*	*24.093*	*0.223*	*LRIG3*	*o*	*1*	*0.167*
*MLL2*	*s*	*20.235*	*0.355*	*LMO1*	*o*	*1*	*0.5*
*CREBBP*	*s/o*	*17.294*	*0.402*	*ASPSCR1*	*o*	*1*	*1*
*ATM*	*s*	*14.404*	*0.4*	*NCOA1*	*o*	*1*	*0.143*
*ARID1A*	*s*	*13.625*	*0.296*	*SRSF2*	*o*	*1*	*0.5*
*NPM1*	*s*	*12.96*	*0.48*	*CBL*	*s/o*	*1*	*0.2*
*EZH2*	*s*	*10.733*	*0.335*	*MYD88*	*o*	*1*	*0.125*
*CIC*	*s*	*10.649*	*0.41*	*NUP98*	*o*	*1*	*0.01*
*ARID2*	*s*	*8.699*	*0.335*	*PTPN11*	*o*	*1*	*0.2*
*ROS1*	*o*	*7.888*	*0.316*	*CDH1*	*s*	*0.999*	*0.062*
*TET2*	*s*	*7.049*	*0.441*	*PER1*	*o*	*0.999*	*0.125*
*WT1*	*s*	*7.027*	*0.502*	*MEN1*	*s*	*0.999*	*0.1*
*CTNNB1*	*o*	*6.862*	*0.114*	*PRDM1*	*s*	*0.999*	*0.125*
*PBRM1*	*s*	*6.854*	*0.685*	*IL21R*	*o*	*0.998*	*0.25*
*PTEN*	*s*	*6.801*	*0.235*	*MUC1*	*o*	*0.997*	*0.332*
*BRAF*	*o*	*5.988*	*0.23*	*PIK3R1*	*s*	*0.997*	*0.066*
*NOTCH2*	*o*	*5.907*	*0.281*	*TRIP11*	*o*	*0.996*	*0.1*
*MYST4*	*o*	*5.492*	*0.25*	*ITK*	*o*	*0.995*	*0.059*
*SMARCA4*	*s*	*5.45*	*0.156*	*NCOA2*	*o*	*0.995*	*0.199*
*DNMT3A*	*s*	*5.293*	*0.23*	*BCOR*	*s*	*0.995*	*0.059*
*ASXL1*	*s*	*5.064*	*0.362*	*TSHR*	*o*	*0.995*	*0.124*
*MYH9*	*o*	*4.988*	*0.416*	*MAP2K4*	*s*	*0.994*	*0.076*
*KDM5A*	*o*	*4.987*	*0.623*	*GNAS*	*o*	*0.994*	*0.062*
*NSD1*	*o*	*4.965*	*0.414*	*MSH2*	*s*	*0.993*	*0.199*
*MYH11*	*o*	*4.561*	*0.198*	*SRGAP3*	*o*	*0.993*	*0.165*
*BCL2*	*o*	*4.165*	*0.116*	*FGFR2*	*o*	*0.992*	*0.142*
*RET*	*o*	*3.991*	*0.333*	*FANCD2*	*s*	*0.991*	*0.33*
*EP300*	*s*	*3.991*	*0.174*	*MET*	*o*	*0.991*	*0.099*
*ALK*	*o*	*3.972*	*0.306*	*EWSR1*	*o*	*0.991*	*0.142*
*PHF6*	*s*	*3.264*	*0.297*	*BRIP1*	*s*	*0.99*	*0.165*
*BLM*	*s*	*3.076*	*0.22*	*SETD2*	*s*	*0.99*	*0.062*
*IDH2*	*o*	*3*	*0.214*	*BCL6*	*o*	*0.989*	*0.11*
*COL1A1*	*o*	*2.997*	*0.231*	*WHSC1*	*o*	*0.985*	*0.123*
*GNA11*	*o*	*2.996*	*0.749*	*BAP1*	*s*	*0.983*	*0.089*
*SF3B1*	*o*	*2.993*	*0.136*	*ETV6*	*o*	*0.953*	*0.136*
*TPR*	*o*	*2.987*	*0.373*	*FLI1*	*o*	*0.902*	*0.18*
*CDH11*	*o*	*2.987*	*0.187*	*DNM2*	*s*	*0.89*	*0.064*
*PDGFRA*	*o*	*2.971*	*0.186*	*BCR*	*o*	*0.888*	*0.296*
*MLL*	*o*	*2.847*	*0.142*	*VHL*	*s*	*0.818*	*0.055*
*KIT*	*o*	*2.59*	*0.173*	*FANCC*	*s*	*0.711*	*0.711*
*MED12*	*o*	*2.514*	*0.168*	*MAF*	*o*	*0.682*	*0.341*
*FBXW7*	*s*	*2.428*	*0.069*	*RUNX1*	*o*	*0.65*	*0.043*
*RB1*	*s*	*2.204*	*0.092*	*CARD11*	*o*	*0.5*	*0.019*
*LIFR*	*o*	*2.175*	*0.128*	*LHFP*	*o*	*0.5*	*0.167*
*AKAP9*	*o*	*2.156*	*0.108*	*NUMA1*	*o*	*0.453*	*0.032*
*NRAS*	*o*	*2.037*	*0.06*	*SUZ12*	*o*	*0.397*	*0.066*
*EGFR*	*o*	*2*	*0.154*	*KIAA1549*	*o*	*0.319*	*0.106*
*JAK2*	*o*	*2*	*0.2*	*NCOA4*	*o*	*0.314*	*0.157*
*PALB2*	*s*	*1.991*	*0.153*	*FHIT*	*o*	*0.257*	*0.086*
*NF1*	*s*	*1.989*	*0.071*	*TNFAIP3*	*s*	*0.254*	*0.023*
*JAK1*	*o*	*1.986*	*0.397*	*NFKB2*	*o*	*0.22*	*0.11*
*PRDM16*	*o*	*1.97*	*0.246*	*MDM4*	*o*	*0.198*	*0.066*
*CAMTA1*	*o*	*1.703*	*0.122*	*H3F3A*	*o*	*0.197*	*0.197*
*NOTCH1*	*o*	*1.681*	*0.06*	*HIP1*	*o*	*0.196*	*0.028*
*CCND2*	*o*	*1.628*	*0.543*	*SUFU*	*s*	*0.188*	*0.047*
*SMARCB1*	*s*	*1.601*	*0.133*	*SBDS*	*s*	*0.184*	*0.092*
*NF2*	*s*	*1.5*	*0.3*	*JAK3*	*o*	*0.144*	*0.013*
*FOXP1*	*o*	*1.5*	*0.15*	*HOOK3*	*o*	*0.11*	*0.026*
*CD74*	*o*	*1.499*	*0.75*	*TCF7L2*	*o*	*0.094*	*0.006*
*CCND3*	*o*	*1.404*	*0.14*	*EIF4A2*	*o*	*0.069*	*0.007*
*PIM1*	*o*	*1.402*	*0.175*	*ERBB2*	*o*	*0.06*	*0.003*
*SLC45A3*	*o*	*1.384*	*0.154*	*ZNF331*	*o*	*0.052*	*0.013*
*IL7R*	*o*	*1.27*	*0.159*	*C15orf55*	*o*	*0.046*	*0.011*
*BTG1*	*o*	*1.174*	*0.196*	*HOXA11*	*o*	*0.045*	*0.015*
*ZNF521*	*o*	*1.148*	*0.057*	*PHOX2B*	*s*	*0.018*	*0.006*
*PDE4DIP*	*o*	*1.099*	*0.061*	*PCM1*	*o*	*0.008*	*0.002*
*MSH6*	*s*	*1.017*	*0.17*	*MALT1*	*o*	*0.003*	*0.001*
*LMO2*	*o*	*1*	*0.5*	**Total**		***1034.4***	

*s*: tumor-suppressor gene, *o*: oncogene. The ratios of the summed frequencies of *s*, *o*, and *s/o* to the total are 0.775, 0.207 and 0.018, respectively.

Rate is the ratio of Times to the gene’s total mutation number in the samples in question.
